# Unusual Presentation with Orbital Mass in a Child with Precursor B-Cell Acute Lymphoblastic Leukemia

**DOI:** 10.1155/2019/8264689

**Published:** 2019-11-05

**Authors:** Lalita Sathitsamitphong, Rungrote Natesirinilkul, Worawut Choeyprasert, Pimlak Charoenkwan

**Affiliations:** Department of Pediatrics, Faculty of Medicine, Chiang Mai University, Chiang Mai, Thailand

## Abstract

Orbital involvement is one of the extramedullary manifestations in acute leukemia. It is common in acute myeloid leukemia, but rare in acute lymphoblastic leukemia (ALL). We described a 3-year-old girl who presented with progressive proptosis of the right eye and was later diagnosed with precursor B-cell ALL. Initial blood count showed Hb 6.9 g/dL, WBC 42,000/mm^3^, lymphoblast 50%, and platelet count 185,000/mm^3^. Bone marrow aspiration revealed 90% lymphoblasts with positivity for CD10, CD19, CD20, CD22, and HLA-DR markers. Computed tomography (CT) scan of the brain and orbit revealed a homogeneous enhancing mass involving the right orbit with intracranial extension. The cytogenetic study showed 46,XX chromosomes. After 4 weeks of induction chemotherapy for very high-risk ALL, although the bone marrow was in remission, the proptosis was partially resolved. CT scan confirmed a decrease in size of the right orbital mass and degree of intracranial extension. Unfortunately, the patient abandoned the treatment after the induction chemotherapy. The actual incidence of orbital involvement in ALL is unknown. Previous case reports describe diverse manifestations of orbital involvement in ALL. The involvement may be unilateral or bilateral, may occur at first diagnosis or at relapse, and may be seen in isolation or with other systemic symptoms. There is no standard treatment protocol. Chemotherapy with or without radiotherapy is generally suggested. The role of upfront hematopoietic stem cell transplantation remains inconclusive. The previously reported prognosis of ALL with orbital involvement is poor.

## 1. Introduction

Acute lymphoblastic leukemia (ALL), the most common malignancy in childhood, is a clonal proliferation of immature lymphoid progenitor cells. Patients with ALL typically present with fever, pallor, bleeding, bone pain, lymphadenopathy, and hepatosplenomegaly which result from the infiltration of the bone marrow and organs by the lymphoblasts. Orbital involvement, which is a common presentation in acute myeloid leukemia (AML), is rare in ALL.

## 2. Case Presentation

A 3-year-old girl presented with painless proptosis of the right eye which progressively developed in ten days prior to admission. She did not have eye discharge, fever, or other systemic symptoms. Physical examination revealed marked proptosis with chemosis of the right eye and limited extraocular movement in all directions. A retrobulbar mass of the right eye was suspected. The left eye was normal. The red reflex of both eyes was present. Ophthalmologic examination showed that the retina and optic discs of both eyes were normal. In addition, the patient was pale and enlarged liver and spleen were noted.

## 3. Materials and Methods

Complete blood count showed hemoglobin 6.9 g/dL, hematocrit 20.7%, white blood cell count 42,000/mm^3^ (lymphoblasts 50%), and platelet count 185,000/mm^3^. Bone marrow aspiration showed 90% lymphoblasts. The immunophenotype analysis by flow cytometry showed positivity for CD10, CD19, CD20, CD22, and HLA-DR markers. Precursor B-cell ALL was diagnosed. Computed tomography (CT) scan of the brain and orbits revealed a 5.0 × 4.2 × 4.9 cm^3^ homogeneously enhancing mass involving superolateral intra- and extraconal components of the right orbit with intracranial extension along the right frontal and temporal convexities. The bone marrow cytogenetic study showed 46, XX chromosomes.

Granulocytic sarcoma or chloroma associated with AML was the provisional diagnosis based on the findings of the orbital mass, hepatosplenomegaly, and blast cells in peripheral blood smear. Other differential diagnoses of benign tumors, such as lymphangioma or dermoid cyst and orbital cellulitis, were less likely with the presence of the blast cells. However, the diagnosis of precursor B-cell ALL was made according to the blast cell morphology and immunophenotype. The patient was categorized as having very high-risk ALL due to the central nervous system involvement.

## 4. Results and Discussion

The patient was given a 4-week induction chemotherapy as per the very high-risk ALL protocol composed of vincristine, prednisolone, doxorubicin, and L-asparaginase. Due to the risk of brain herniation, the intrathecal methotrexate was initially deferred and given on the extended 5^th^ and 6^th^ week of induction chemotherapy. Cranial irradiation was planned upon the completion of induction chemotherapy.

**Figure 1:**
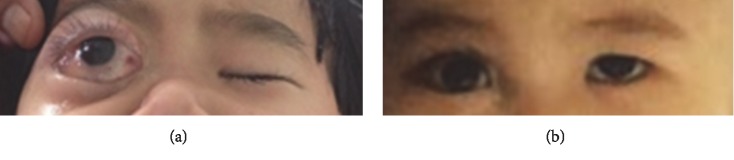
(a) Photograph demonstrating proptosis of the right eye. (b) After 4 weeks of induction chemotherapy, decrease in the size of proptosis.

**Figure 2:**
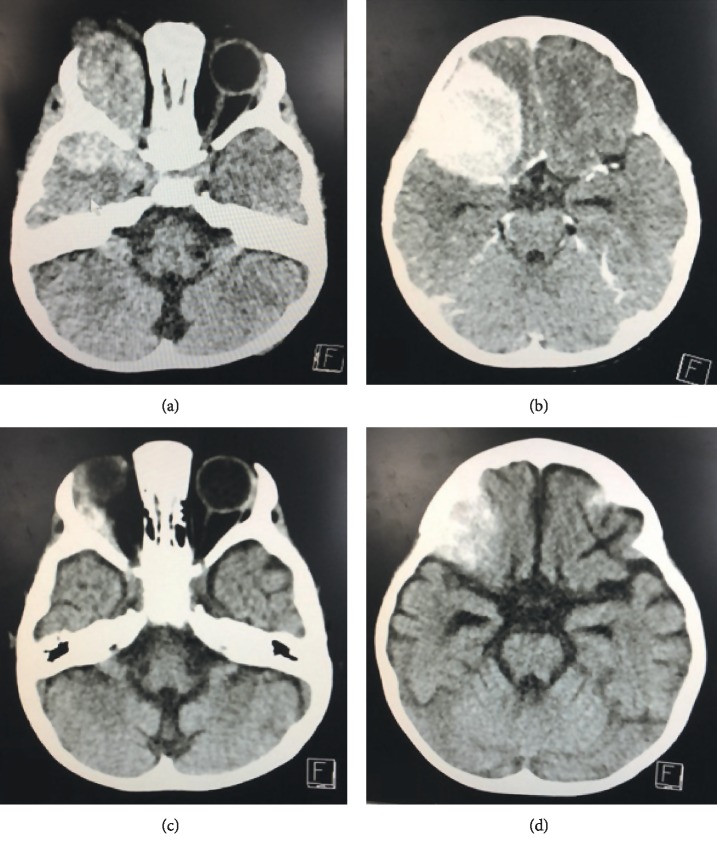
(a, b) Computed tomography scan revealed a homogeneous enhancing mass involving the right orbit with intracranial extension. (c, d) After 4 weeks of induction chemotherapy, decrease in the size of homogeneous enhancing mass involving the right orbit with intracranial extension.

After 4 weeks of induction chemotherapy, physical examination showed a decrease in size of proptosis up to 75% and no enlarged liver and spleen (Figure 1). Bone marrow aspiration revealed bone marrow in remission with normal trilineage hematopoiesis. CT scan of the brain and orbits showed a decrease in the orbital mass size measuring 2.9 × 2.9 × 2.7 cm^3^ (Figure 2). Cerebrospinal fluid analysis after 4-week induction chemotherapy revealed no malignant cells. Unfortunately, because of socioeconomic restraints, the patient abandoned the treatment after the induction chemotherapy was completed.

Ophthalmic involvement which consists of orbital and ocular infiltrations is one of the extramedullary manifestations of acute leukemia. While orbital mass is commonly seen in AML and is known as granulocytic sarcoma, it is uncommon in ALL [1]. In a study of 1,264 patients with orbital tumors, 130 were classified as having lymphoid or leukemic lesions. Only two patients were diagnosed with ALL [2].

There are sporadic case reports of patients with ALL who present with an orbital mass, either as an isolated manifestation or seen concomitantly with other systemic manifestations [1, 3–6]. The treatment mostly consists of systemic chemotherapy with or without orbital and craniospinal irradiation [1, 3–9]. The outcomes vary from complete remission after systemic chemotherapy without radiotherapy to relapse and death after chemotherapy and radiotherapy [1, 3–9]. Bidar et al. reported a successful treatment in three patients with hematopoietic stem cell transplantation (HSCT) without radiotherapy [7]. To date, there is no standard treatment protocol for ALL with orbital mass.

Ophthalmic involvement could also be the manifestation of relapsed ALL. Tabata et al. reported a 9-year-old boy who was diagnosed with relapsed ALL and developed an orbital mass post matched unrelated HSCT. The patient achieved engraftment but developed a right orbital mass on day 21 after HSCT and later progressed to systemic relapse [8]. Tayler et al. reported three relapsed ALL cases with ophthalmic involvement, consisted of proptosis, visual acuity impairment, hypopyon, and papilledema. The ophthalmic involvement was bilateral in one patient. All three received chemotherapy and radiotherapy. Two patients achieved complete remission but one had cataract and one had permanent visual acuity impairment [9].

The prognosis in ALL patients with ophthalmic involvement is generally poor. From four previous reports, five of 14 ALL patients with ophthalmic involvement (35.7%) died of disease [6–9]. Three of five patients had ALL at the first diagnosis and two had relapsed ALL [6, 7]. The role of upfront HSCT remains inconclusive. Five of 10 ALL cases at the first diagnosis with orbital involvement attained complete remission without HSCT [1, 3–5, 7]. Among three patients with first-diagnosed ALL who underwent HSCT, one patient died [7]. Another two reports showed that two out of four relapsed ALL cases with orbital involvement who were treated with chemotherapy and did not undergo HSCT were able to achieve remission [8, 9]. Further studies are required to establish the treatment strategies in ALL patients with orbital involvement to improve the outcome and long-term survival.

## 5. Conclusion

Orbital involvement is an uncommon extramedullary manifestation of ALL, and the actual incidence is unknown. The manifestations of orbital involvement in ALL are diverse. The involvement may be unilateral or bilateral, may occur at first diagnosis or at relapse, and may be seen in isolation or with other systemic symptoms. The prognosis of ALL with orbital involvement is poor. Systemic chemotherapy with or without radiotherapy should be the principal treatment in the first-diagnosed ALL patients with orbital involvement. Radiotherapy may be omitted in patients who achieve complete response to chemotherapy with no residual orbital mass. The role of HSCT may be considered in both relapse ALL patients with orbital involvement and first-diagnosed patients who could not achieve complete response to systemic chemotherapy and radiotherapy. HSCT may improve the treatment outcomes in these patients.
